# A mathematical model of the sterol regulatory element binding protein 2 cholesterol biosynthesis pathway

**DOI:** 10.1016/j.jtbi.2014.01.013

**Published:** 2014-05-21

**Authors:** Bonhi S. Bhattacharya, Peter K. Sweby, Anne-Marie Minihane, Kim G. Jackson, Marcus J. Tindall

**Affiliations:** a Department of Mathematics and Statistics, University of Reading, Whiteknights, Reading RG6 6AX, UK; b Department of Nutrition, Norwich Medical School, University of East Anglia, Norwich NR4 7TJ, UK; c Department of Food and Nutritional Sciences, University of Reading, Whiteknights, Reading RG6 6AP, UK; d School of Biological Sciences, University of Reading, Whiteknights, Reading RG6 6AJ, UK; e Institute of Cardiovascular and Metabolic Research, University of Reading, Whiteknights, Reading RG6 6AA, UK

**Keywords:** Genetic regulation, Transcription factor, Nonlinear ordinary differential equation, SREBP-2

## Abstract

Cholesterol is one of the key constituents for maintaining the cellular membrane and thus the integrity of the cell itself. In contrast high levels of cholesterol in the blood are known to be a major risk factor in the development of cardiovascular disease. We formulate a deterministic nonlinear ordinary differential equation model of the sterol regulatory element binding protein 2 (SREBP-2) cholesterol genetic regulatory pathway in a hepatocyte. The mathematical model includes a description of genetic transcription by SREBP-2 which is subsequently translated to mRNA leading to the formation of 3-hydroxy-3-methylglutaryl coenzyme A reductase (HMGCR), a main regulator of cholesterol synthesis. Cholesterol synthesis subsequently leads to the regulation of SREBP-2 via a negative feedback formulation. Parameterised with data from the literature, the model is used to understand how SREBP-2 transcription and regulation affects cellular cholesterol concentration. Model stability analysis shows that the only positive steady-state of the system exhibits purely oscillatory, damped oscillatory or monotic behaviour under certain parameter conditions. In light of our findings we postulate how cholesterol homeostasis is maintained within the cell and the advantages of our model formulation are discussed with respect to other models of genetic regulation within the literature.

## Highlights

• We formulate and analyse a nonlinear ODE model of the SREBP2 pathway.
• The mathematical model exhibits stable limit cycles under certain parameter conditions.
• Negative feedbacks in the SREBP2 pathway may help regulate cholesterol homeostasis.
• Our model provides a more accurate formulation of genetic regulation using nonlinear ODEs.

## 1 Introduction and motivation

As an essential constituent of the plasma membrane of mammalian cells, cholesterol is used for the maintenance of both membrane structural integrity and selective permeability (Simons and Iknonen, 2000). However, superfluous cholesterol levels result in cellular toxicity (Yeagle, 1991; Tabas, 1997; Tangirala et al., 1994). Insufficient cholesterol causes cytotoxicity via compromised membrane structure. Furthermore cellular cholesterol metabolism is a key modulator of plasma cholesterol, with the management of plasma hypercholesterolaemia at the cornerstone of population cardiovascular disease management (Grundy et al., 2004). It is therefore crucial that intracellular cholesterol levels are strictly regulated. Cellular cholesterol homeostasis, the property to maintain cholesterol concentration to within narrow ranges, results from a balance of three mechanisms: efflux, influx and biosynthesis.

Understanding the mechanisms which regulate cellular cholesterol content is vital to understanding pathology associated with sub- and supra-optimal cell and blood cholesterol concentrations. These levels are dependent on both the balance between dietary cholesterol intake and *de novo* synthesis of cholesterol within cells.

The low density lipoprotein receptor (LDLR) protein forms part of the lipoprotein metabolic pathway responsible for the clearance of cholesterol from the circulation (Brown and Goldstein, 1979; Goldstein et al., 1985). Biosynthesis of cholesterol is a multistep reaction in which the rate-limiting step is the reduction of 3-hydroxy-3-methylglutaryl coenzyme A (HMG-CoA) in the reaction catalysed by the enzyme HMG-CoA reductase (HMGCR).

Over accumulation or excessive depletion of free cholesterol within the cell is prevented by a negative feedback loop that responds to elevations or depressions in intracellular cholesterol. This feedback loop exerts the majority of its control by regulating the synthesis of the two key proteins: HMGCR and LDLR. In brief, when the intracellular cholesterol level is low, both LDLR and HMGCR synthesis are activated, thereby increasing the influx of cholesterol via the LDLR pathway, and the biosynthesis of cholesterol in the cell. If conversely there are high cholesterol levels in the cell, synthesis of LDLR and HMGCR declines.

There has been much research conducted into the response of cell cholesterol to dietary intake, with the dietary fatty acid composition rather than cholesterol intake reported to have a greater impact on circulating cholesterol concentrations. In particular, partial replacement of saturated fat with either monounsaturated (found in olive oil) or *n*-6 polyunsaturated (found in vegetable oils such as sunflower oil) fatty acids have been associated with significant reductions in both total and LDL-cholesterol concentrations (Mensink et al., 2003; Micha and Mozaffarian, 2010). Dietary fat composition is considered to influence circulating cholesterol concentrations via effects on hepatic cholesterol synthesis and the expression of genes involved in circulating LDL-cholesterol metabolism (Xu et al., 1999).

Previous mathematical modelling has included compartmental models of the lipoprotein metabolic pathway (Knoblauch et al., 2000; Packard et al., 2000; Adiels et al., 2005) and dynamic models of lipoprotein metabolism in conjunction with the LDLR pathway (August et al., 2007; Wattis et al., 2008). Of particular note in these dynamic models is the lack of explicit representation of the cholesterol biosynthesis reaction and as a consequence, the interplay between cholesterol biosynthesis, the LDLR uptake of lipoprotein cholesterol and cholesterol mediated negative feedback is not fully appreciated.

The cholesterol biosynthetic pathway is already the basis of the most common form of pharmaceutical treatment for high plasma cholesterol levels. HMGCR inhibitors, more commonly known as statins, act as competitive inhibitors of the HMGCR enzyme. By inhibiting the biosynthesis of cholesterol, statins deplete intracellular cholesterol concentration and promote the synthesis of both HMGCR and the LDLR, thereby increasing the uptake of lipoproteins (and plasma cholesterol) via the LDLR. It is recognised that individual response to statin treatment varies widely. Genetic variation in *HMGCR* has been associated with a diminished lipid lowering response (Chasman et al., 2004; Krauss et al., 2008), suggesting that the cholesterol biosynthetic pathway plays an important role in the control of plasma cholesterol levels.

However, relatively little modelling has been conducted to investigate the qualitative behaviour of the processes which govern *de novo* cholesterol synthesis at a genetic level, which may provide a better understanding of such phenomena. The mathematical model presented in this paper will examine the underlying genetic mechanisms governing cholesterol biosynthesis as a first step towards elucidating the dynamics of this pathway.

The paper is organised as follows. In Section 2 the biological processes which describe the genetic regulation of cholesterol biosynthesis are reviewed. Following this, the mathematical model is derived in Section 3 and details of model parameter values obtained from the literature are summarised in Section 4. Model analysis is undertaken in Sections 5–7 and the results are summarised and discussed in Section 8.

## 2 Regulated expression of cholesterol biosynthetic genes

**Fig. 1:**
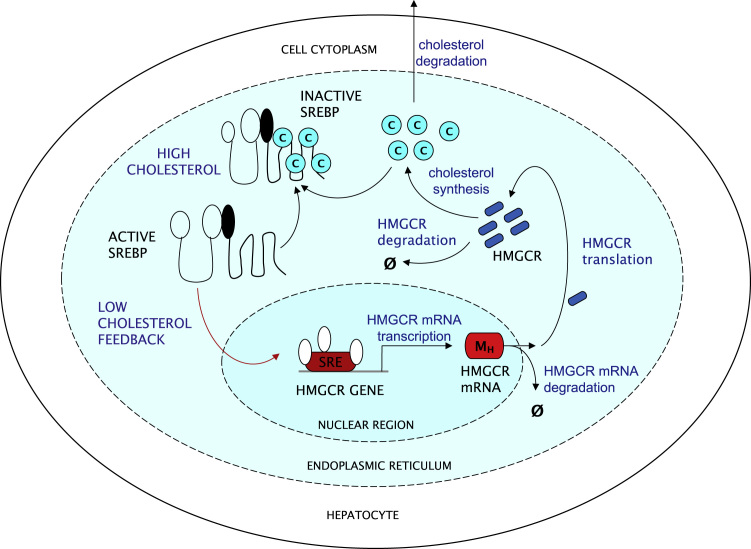
Genetic regulation of cholesterol biosynthesis by SREBP-2. Hepatocytes synthesise HMGCR mRNA which in turn is translated into the enzyme HMGCR. HMGCR catalyses the synthesis of cholesterol which in turn influences its own transcription rate by interacting with the transcription factor SREBP; the transcription rate increases when cholesterol is low in the cell and declines when cholesterol is high. (SRE – sterol regulatory element; M_H_ – HMGCR mRNA; C – cholesterol).

A major point of control of the cholesterol biosynthetic pathway occurs at the level of gene expression in response to cellular cholesterol levels, as shown in Fig. 1. The insolubility of cholesterol dictates that it cannot directly influence a genetic response. The critical role in controlling the expression of a range of genes involved in the regulation of cellular lipid homeostasis falls to the three isoforms of the SREBP family of transcription factors, SREBP-1a, SREBP-1c and SREBP-2. In particular, the SREBP-2 isoform is relatively specific to regulating the expression of many enzymes involved in cholesterol biosynthesis (Brown and Goldstein, 1997).

SREBPs exist normally in a tight complex with the SREBP cleavage activating protein (SCAP) within the endoplasmic reticulum of cells. SCAP consists of two domains, one of which is responsible for the association with SREBP. The other domain contains a region known as the sterol sensing domain (SSD). When the cellular cholesterol concentration becomes depleted, SCAP escorts SREBP to the Golgi apparatus of the cell, where it undergoes sequential cleavage by proteases. The net effect of this is to liberate the transcription factor, nuclear SREBP which can then enter the cell nucleus (Eberlé et al., 2004). Here it binds to a regulatory binding site (a short sequence of DNA) on the promoter region of the target gene known as the sterol regulatory element (SRE) and activates its transcription (Soutar and Knight, 1990).

In the presence of replete cellular sterol concentrations, cholesterol binds directly to the SSD of SCAP. This causes a conformational change in SCAP which results eventually in the anchoring of the SCAP–SREBP complex to the endoplasmic reticulum (ER) membrane (Yang et al., 2002). This process is responsible for the retention of the SCAP–SREBP complex within the ER. Transcription of the target genes declines.

In the context of the HMGCR gene, when a cell's cholesterol levels are low, the SCAP–SREBP complex is active and free to move. In such a state SREBP is formed and is able to reach the nucleus and activate HMGCR mRNA transcription and thus HMGCR synthesis, increasing the cholesterol concentration in the cell by upregulating its synthesis. If, conversely, there are high cellular cholesterol levels, then SCAP–SREBP is unable to move and effectively inactive. Consequently both HMGCR mRNA transcription and HMGCR translation decrease, and cholesterol synthesis is reduced.

In a simplified model of the gene expression response to cellular cholesterol concentration, the system can be seen as an end product negative feedback loop system, in the manner of the mathematical models of expression developed by, for example, Goodwin (1963, 1965) and Griffith (1968). In such models, the response of the gene is directly dependent upon the concentration of cholesterol. A very low level of cholesterol will provoke a large response in the synthesis of HMGCR enzyme, and vice-a-versa. Theoretically, this results in a considerable range over which the model allows cholesterol concentration to vary. This is, however, uncharacteristic of the homeostatic property which the physiological system possesses, and which ensures that cellular cholesterol can only fluctuate within a narrow range of values, to avoid the cytotoxicity associated with extreme values.

The addition of the SREBP transcription factor function models the underlying biological mechanism, and also introduces complexity to the negative feedback loop in the form of an activator function which is suppressed by accumulation of an end product. In the following section a model of this interaction between SREBP and cholesterol, and the effect on gene expression are presented.

## 3 The model

The interactions characterising cellular cholesterol homeostasis and its regulation by transcription factors are many, and a full model of all variables and reactions is not necessarily pragmatic. Furthermore, the number of parameter values required will increase with complexity. Previous models have shown that excessive simplification can fail to reproduce dynamics which have been observed in experimental settings.

As an example, the work by Wattis et al. (2008) models non-lipoprotein cholesterol influx to the cell as proportional to the difference between cell cholesterol concentration and a predetermined ideal equilibrium value; this produces the correct dynamics for cell cholesterol response. An interesting consequence of this formalism, though, is that intracellular cholesterol concentration in the model reaches equilibrium rapidly (on a timescale of the order of minutes) after an influx of lipoprotein cholesterol to the cell. However, experimental results suggest that this may not be the case, with changes in intracellular cholesterol concentration occurring on timescales of 12–24 h (Liscum and Faust, 1987; Liscum et al., 1989). This suggests that not enough complexity is included here to capture the longer term dynamics of cholesterol synthesis at the level of the HMGCR gene.

A further requirement is that the system must respond naturally in the absence or presence of cholesterol as opposed to only acting reasonably under certain circumstances. For example, in the work of August et al. (2007), all cholesterol in the cell is assumed to be derived from lipoprotein sources. Whilst this reproduces the required qualitative behaviour under the conditions whereby extracellular lipoprotein is present, in the case where this is zero, the intracellular cholesterol level falls to zero, which is physiologically fatal for the cell.

The work presented in this paper is focused on formulating and analysing a nonlinear ordinary differential equation (ODE) model of the SREBP-2 cholesterol biosynthesis pathway. The goal of the work is to understand cholesterol regulation via the negative feedback between SREBP-2 transcription and cholesterol and to what extent this affects the steady-state cholesterol levels of the cell. In doing so we hope to more accurately capture cellular regulation of cholesterol and be able to understand it in the wider context of dietary cholesterol intake.

### 3.1 Mathematical model formulation

**Fig. 2:**
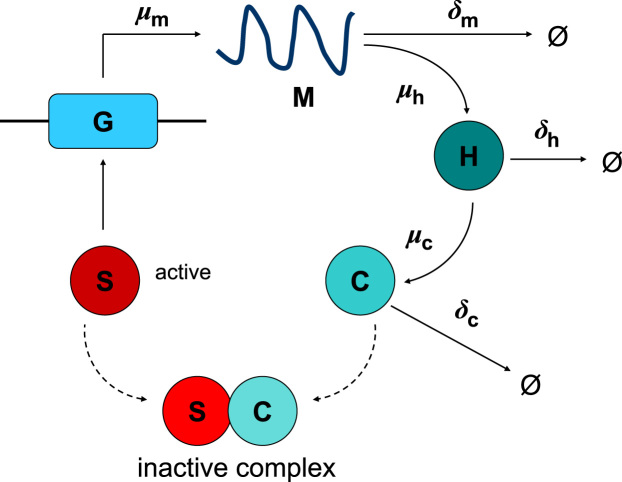
The genetic regulation of cholesterol production by SREBP-2. The HMGCR gene $\overset{¯}{\text{G}}$ is transcribed at a rate ${\overset{¯}{\mu }}_{m}$ to produce HMGCR mRNA $\overset{¯}{\text{M}}$. This is translated at a rate ${\overset{¯}{\mu }}_{h}$ into the HMGCR enzyme $\overset{¯}{\text{H}}$. HMGCR then goes on to catalyse the reaction creating the metabolite cholesterol $\overset{¯}{\text{C}}$ at a rate ${\overset{¯}{\mu }}_{c}$. This process is under the control of the transcription factor SREBP $\overset{¯}{\text{S}}$ which acts as a transcriptional activator for the pathway. Under conditions where cholesterol $\overset{¯}{\text{C}}$ is in excess $\overset{¯}{\text{S}}$ forms an inactive complex with $\overset{¯}{\text{C}}$ and transcription of the target gene declines. HMGCR mRNA, HMGCR and cholesterol are degraded at rates ${\overset{¯}{\delta }}_{m}$, ${\overset{¯}{\delta }}_{h}$ and ${\overset{¯}{\delta }}_{c}$, respectively.

In this section we derive a system of nonlinear ODEs to describe the genetic regulation of cholesterol biosynthesis by SREBP-2 as summarised in Fig. 2.

The binding of SREBP-2 to the gene, subsequent transcription and translation to HMG-CoA mRNA and production of HMGCR and cholesterol can be described by the reaction equation(1)

Here *x* is the number of molecules of $\overset{¯}{\text{S}}$ required to bind to $\overset{¯}{\text{G}}$ to produce a functional effect. This binding reaction has an association rate ${\overset{¯}{\kappa }}_{1}$ and a dissociation rate ${\overset{¯}{\kappa }}_{-1}$. $\overset{¯}{\text{M}}$ is transcribed at a rate ${\overset{¯}{\mu }}_{d}$ and $\overset{¯}{\text{H}}$ is translated at a rate ${\overset{¯}{\mu }}_{h}$. The creation of $\overset{¯}{\text{C}}$ occurs at a rate ${\overset{¯}{\mu }}_{c}$. ${\overset{¯}{\delta }}_{m}$, ${\overset{¯}{\delta }}_{h}$ and ${\overset{¯}{\delta }}_{c}$ are respectively the degradation rates of $\overset{¯}{\text{M}}$, $\overset{¯}{\text{H}}$ and $\overset{¯}{\text{C}}$.

Similarly the binding of cholesterol to active SREBP-2 to form an inactive complex which down-regulates the transcription of cholesterol (negative feedback) is given by

(2) $$\overset{¯}{\text{S}}+y\overset{¯}{\text{C}}\overset{{\overset{¯}{\kappa }}_{2}}{\underset{{\overset{¯}{\kappa }}_{-2}}{⇄}}\overset{¯}{\text{S}}:y\overset{¯}{\text{C}},$$

where *y* is the number of molecules of $\overset{¯}{\text{C}}$ required to bind to $\overset{¯}{\text{S}}$ to cause inactivation. This binding reaction has an association rate ${\overset{¯}{\kappa }}_{2}$ and a dissociation rate ${\overset{¯}{\kappa }}_{-2}$.

We note two important biological concepts arising from the physiological mechanism of gene expression or protein synthesis, which will affect the form of the ODEs (Alberts et al., 2008) describing Eqs. (1) and (2).

(i) $\left[\overset{¯}{\text{G}}:x\overset{¯}{\text{S}}\right]$ represents the concentration of DNA in an active state, which is able to undergo transcription. During transcription, activated DNA is copied by the action of an enzyme to produce mRNA. This process does not deplete $\left[\overset{¯}{\text{G}}:x\overset{¯}{\text{S}}\right]$.
(ii) Protein is synthesised from mRNA via the action of ribosomes. Following protein synthesis, mRNA detaches from the ribosome and the mRNA is free to participate in further synthesis reactions until it is degraded according to its half-life. Therefore, the synthesis of the enzyme, $\overset{¯}{\text{H}}$, does not affect the concentration of $\overset{¯}{\text{M}}$. That is, synthesis of $\overset{¯}{\text{H}}$ will *not* deplete $\overset{¯}{\text{M}}$.

The governing ODEs equations are derived by application of the law of mass action to the biochemical reactions (1) and (2) which gives

(3) $$\frac{d\overset{¯}{g}}{\mathrm{dt}}={\overset{¯}{\kappa }}_{-1}{\overset{¯}{s}}_{b}-{\overset{¯}{\kappa }}_{1}{\overset{¯}{s}}^{\;x}\overset{¯}{g},$$

(4) $$\frac{d\overset{¯}{s}}{\mathrm{dt}}=x{\overset{¯}{\kappa }}_{-1}{\overset{¯}{s}}_{b}-x{\overset{¯}{\kappa }}_{1}{\overset{¯}{s}}^{\;x}\overset{¯}{g}-{\overset{¯}{\kappa }}_{2}{\overset{¯}{c}}^{\;y}\overset{¯}{s}+{\overset{¯}{\kappa }}_{-2}{\overset{¯}{c}}_{b},$$

(5) $$\frac{d{\overset{¯}{s}}_{b}}{\mathrm{dt}}=-{\overset{¯}{\kappa }}_{-1}{\overset{¯}{s}}_{b}+{\overset{¯}{\kappa }}_{1}{\overset{¯}{s}}^{\;x}\overset{¯}{g},$$

(6) $$\frac{d\overset{¯}{m}}{\mathrm{dt}}={\overset{¯}{\mu }}_{d}{\overset{¯}{s}}_{b}-{\overset{¯}{\delta }}_{m}\overset{¯}{m},$$

(7) $$\frac{d\overset{¯}{h}}{\mathrm{dt}}={\overset{¯}{\mu }}_{h}\overset{¯}{m}-{\overset{¯}{\delta }}_{h}\overset{¯}{h},$$

(8) $$\frac{d\overset{¯}{c}}{\mathrm{dt}}={\overset{¯}{\mu }}_{c}\overset{¯}{h}+y{\overset{¯}{\kappa }}_{-2}{\overset{¯}{c}}_{b}-{\overset{¯}{\delta }}_{c}\overset{¯}{c}-y{\overset{¯}{\kappa }}_{2}{\overset{¯}{c}}^{\;y}\overset{¯}{s},$$

(9) $$\frac{d{\overset{¯}{c}}_{b}}{\mathrm{dt}}={\overset{¯}{\kappa }}_{2}{\overset{¯}{c}}^{\;y}\overset{¯}{s}-{\overset{¯}{\kappa }}_{-2}{\overset{¯}{c}}_{b},$$

with initial conditions

(10) $$\overset{¯}{g}(0)={\overset{¯}{g}}_{0},\;\overset{¯}{s}(0)={\overset{¯}{s}}_{0},\;{\overset{¯}{s}}_{b}(0)=0,\;\overset{¯}{m}(0)={\overset{¯}{m}}_{0},\overset{¯}{h}(0)={\overset{¯}{h}}_{0},\;\overset{¯}{c}(0)={\overset{¯}{c}}_{0},\;{\overset{¯}{c}}_{b}(0)=0,$$

where in the above system of equations, we use the following notation in which square brackets denote concentration: $\overset{¯}{g}=[\overset{¯}{\text{G}}]$, $\overset{¯}{s}=[\overset{¯}{\text{S}}]$, ${\overset{¯}{s}}_{b}=[\overset{¯}{\text{G}}:x\overset{¯}{\text{S}}]$, $\overset{¯}{m}=[\overset{¯}{\text{M}}]$, $\overset{¯}{h}=[\overset{¯}{\text{H}}]$, $\overset{¯}{c}=[\overset{¯}{\text{C}}]$ and ${\overset{¯}{c}}_{b}=[\overset{¯}{\text{S}}:y\overset{¯}{\text{C}}]$.

The coefficient *x* in the first term of Eq. (4) reflects that the dissociation of one active DNA complex releases *x* molecules of unbound transcription factor. The coefficient *x* in the second term of Eq. (4) states that the creation of one active DNA complex requires up to *x* DNA binding sites.

The number of genes within a cell is constant so adding Eqs. (3) and (5) leads to

(11) $$\frac{d\overset{¯}{g}}{\mathrm{dt}}+\frac{d{\overset{¯}{s}}_{b}}{\mathrm{dt}}=0\Rightarrow \overset{¯}{g}(t)+{\overset{¯}{s}}_{b}(t)={\overset{¯}{g}}_{0},$$

on using the initial conditions (10). We now assume that Eq. (5) reaches equilibrium rapidly (quasi-steady-state approximation) such that

(12) $$\frac{d{\overset{¯}{s}}_{b}}{\mathrm{dt}}\approx 0,$$

and using Eq. (11) we have

(13) $${\overset{¯}{\kappa }}_{1}{\overset{¯}{s}}^{\;x}({\overset{¯}{g}}_{0}-{\overset{¯}{s}}_{b})+{\overset{¯}{\kappa }}_{-1}{\overset{¯}{s}}_{b}≃0,$$

which upon rearranging gives

(14) $${\overset{¯}{s}}_{b}≃\frac{{\overset{¯}{g}}_{0}{\overset{¯}{s}}^{\;x}}{{\overset{¯}{\kappa }}_{m}^{\;x}+{\overset{¯}{s}}^{\;x}},$$

where

(15) $${\overset{¯}{\kappa }}_{m}={\left({\overset{¯}{\kappa }}_{d}\right)}^{1/x}={\left({\overset{¯}{\kappa }}_{-1}/{\overset{¯}{\kappa }}_{1}\right)}^{1/x}.$$

Here ${\overset{¯}{\kappa }}_{d}$ is the dissociation constant for the reaction between $\overset{¯}{\text{S}}$ and $\overset{¯}{\text{G}}$.

We further observe that adding Eqs. (4), (5) and (9) gives

(16) $$\frac{d}{\mathrm{dt}}(\overset{¯}{s}+{\overset{¯}{s}}_{b}+{\overset{¯}{c}}_{b})=(1-x)(-{\overset{¯}{\kappa }}_{-1}{\overset{¯}{s}}_{b}+{\overset{¯}{\kappa }}_{1}{\overset{¯}{s}}^{\;x}g),$$

(17) $$=(1-x)\frac{d{\overset{¯}{s}}_{b}}{\mathrm{dt}}.$$

Under the quasi-steady state assumption of Eq. (12) together with the initial conditions (10) we find that

(18) $$\frac{d}{\mathrm{dt}}(\overset{¯}{s}+{\overset{¯}{s}}_{b}+{\overset{¯}{c}}_{b})\approx 0,$$

(19) $$\Rightarrow \overset{¯}{s}+{\overset{¯}{s}}_{b}+{\overset{¯}{c}}_{b}={\overset{¯}{s}}_{0}.$$

Also under the approximation (12) we see that ${\overset{¯}{s}}_{b}≃{\overset{¯}{s}}_{b}(0)≃0$. This is a valid assumption if we consider that the concentration of binding sites for a particular transcription factor on one particular gene is extremely small compared to the concentration of free transcription factor available in the cell, i.e. ${\overset{¯}{s}}_{b}<<\overset{¯}{s}$. We then obtain the following equation from (19):

(20) $$\overset{¯}{s}+{\overset{¯}{c}}_{b}≃{\overset{¯}{s}}_{0}.$$

Finally we assume that the binding reaction between $\overset{¯}{\text{S}}$ and $\overset{¯}{\text{C}}$ reaches equilibrium rapidly such that

(21) $${\overset{¯}{\kappa }}_{2}{\overset{¯}{c}}^{\;y}\overset{¯}{s}-{\overset{¯}{\kappa }}_{-2}({\overset{¯}{s}}_{0}-\overset{¯}{s})≃0.$$

Rearranging this result gives

(22) $$\overset{¯}{s}=\frac{{\overset{¯}{s}}_{0}}{1+{\left(\overset{¯}{c}/{\overset{¯}{\kappa }}_{c}\right)}^{y}}$$

in which we define the constant ${\overset{¯}{\kappa }}_{c}$ such that

(23) $${\overset{¯}{\kappa }}_{c}={\left({\overset{¯}{\kappa }}_{s}\right)}^{\;1/y}={\left({\overset{¯}{\kappa }}_{-2}/{\overset{¯}{\kappa }}_{2}\right)}^{1/y},$$

where ${\overset{¯}{\kappa }}_{s}$ is the dissociation constant for the reaction between $\overset{¯}{\text{S}}$ and $\overset{¯}{\text{C}}$.

Using Eqs. (14), (20) and (22) to eliminate Eqs. (3)–(5) and (9) from the system equations (3)–(9) we obtain the reduced system

(24) $$\frac{d\overset{¯}{m}}{\mathrm{dt}}=\frac{{\overset{¯}{\mu }}_{m}}{1+{\left(\frac{{\overset{¯}{\kappa }}_{m}(1+{\left(\overset{¯}{c}/{\overset{¯}{\kappa }}_{c}\right)}^{y})}{{\overset{¯}{s}}_{0}}\right)}^{x}}-{\overset{¯}{\delta }}_{m}\overset{¯}{m}=\overset{¯}{f}(\overset{¯}{m},\overset{¯}{h},\overset{¯}{c}),$$

(25) $$\frac{d\overset{¯}{h}}{\mathrm{dt}}={\overset{¯}{\mu }}_{h}\overset{¯}{m}-{\overset{¯}{\delta }}_{h}\overset{¯}{h}=\overset{¯}{g}(\overset{¯}{m},\overset{¯}{h},\overset{¯}{c}),$$

(26) $$\frac{d\overset{¯}{c}}{\mathrm{dt}}={\overset{¯}{\mu }}_{c}\overset{¯}{h}-{\overset{¯}{\delta }}_{c}\overset{¯}{c}=\overset{¯}{j}(\overset{¯}{m},\overset{¯}{h},\overset{¯}{c}),$$

with the initial conditions

(27) $$\overset{¯}{m}(0)={\overset{¯}{m}}_{0},\;\overset{¯}{h}(0)={\overset{¯}{h}}_{0}\;\mathrm{and}\;\overset{¯}{c}(0)={\overset{¯}{c}}_{0}.$$

Here ${\overset{¯}{\mu }}_{m}={\overset{¯}{\mu }}_{d}{\overset{¯}{g}}_{0}$ where ${\overset{¯}{\mu }}_{m}$ is the maximal rate of transcription.

*Non-dimensionalisation*: Before proceeding to a complete analysis of the model, Eqs. (24)–(26) are non-dimensionalised. Time is scaled with respect to the synthesis rate of $\overset{¯}{m}$ such that

(28) $$\tau ={\overset{¯}{\mu }}_{h}t,$$

where *τ* represents the non-dimensional time. The remaining variables are rescaled with respect to the concentration of total transcription factor, ${\overset{¯}{s}}_{0}$, such that

(29) $$m=\frac{\overset{¯}{m}}{{\overset{¯}{s}}_{0}},\;h=\frac{\overset{¯}{h}}{{\overset{¯}{s}}_{0}},\;\mathrm{and}\;c=\frac{\overset{¯}{c}}{{\overset{¯}{s}}_{0}}.$$

This non-dimensionalisation leads to

(30) $$\frac{\mathrm{dm}}{d\tau }=\frac{\mu_{m}}{1+{\left(\kappa_{m}(1+{\left(c/\kappa_{c}\right)}^{y})\right)}^{x}}-\delta_{m}m=f(m,h,c),$$

(31) $$\frac{\mathrm{dh}}{d\tau }=m-\delta_{h}h=g(m,h,c),$$

(32) $$\frac{\mathrm{dc}}{d\tau }=\mu_{c}h-\delta_{c}c=j(m,h,c),$$

with the initial conditions

(33) $$m_{0}=\frac{{\overset{¯}{m}}_{0}}{{\overset{¯}{s}}_{0}},\;h_{0}=\frac{{\overset{¯}{h}}_{0}}{{\overset{¯}{s}}_{0}},\;c_{0}=\frac{{\overset{¯}{c}}_{0}}{{\overset{¯}{s}}_{0}},$$

where the non-dimensional parameters are given by

(34) $$\mu_{m}=\frac{{\overset{¯}{\mu }}_{m}}{{\overset{¯}{\mu }}_{h}{\overset{¯}{s}}_{0}},\;\mu_{c}=\frac{{\overset{¯}{\mu }}_{c}}{{\overset{¯}{\mu }}_{h}},\;\kappa_{m}=\frac{{\overset{¯}{\kappa }}_{m}}{{\overset{¯}{s}}_{0}},\kappa_{c}=\frac{{\overset{¯}{\kappa }}_{c}}{{\overset{¯}{s}}_{0}},\;\delta_{c}=\frac{{\overset{¯}{\delta }}_{c}}{{\overset{¯}{\mu }}_{h}},\;\delta_{h}=\frac{{\overset{¯}{\delta }}_{h}}{{\overset{¯}{\mu }}_{h}},\;\delta_{m}=\frac{{\overset{¯}{\delta }}_{m}}{{\overset{¯}{\mu }}_{h}}.$$

The non-dimensional parameter values are summarised in Table 2.

## 4 Parameter estimation

A summary of the model parameter values is provided in Table 1 with details on how each was derived from the experimental literature given in Appendix A. Wherever possible data elicited from human liver cells (Hep G2) have been used. However, it has not been possible to determine all required parameters in this manner. In some cases the model parameters do not have a direct physiological counterpart since the biological processes occurring have been simplified in the mathematical modelling to reduce complexity; in others, the parameter value is not customarily measured in the required units, not least because of the difficulty in isolating the biosynthesis pathway. In these instances underlying biological principles have been used to estimate a realistic value, and to ensure that the model operates within a plausible physiologic domain.

## 5 Model analysis

In this section and continuing in Sections 6 and 7 we discuss the existence of steady-states of Eqs (30)–(32) and their stability.

### 5.1 Fixed point analysis

The steady states of equations (30)–(32) are given by the solution of

(35) $$0=\frac{\mu_{m}}{1+{\left(\kappa_{m}(1+{\left(c_{\mathrm{ss}}/\kappa_{c}\right)}^{y})\right)}^{x}}-\delta_{m}m_{\mathrm{ss}},$$

(36) $$0=m_{\mathrm{ss}}-\delta_{h}h_{\mathrm{ss}},$$

(37) $$0=\mu_{c}h_{\mathrm{ss}}-\delta_{c}c_{\mathrm{ss}},$$

where *m*_*ss*_, *h*_*ss*_ and *c*_*ss*_ are the steady state values of *m*, *h* and *c* respectively. Using Eqs. (36) and (37), Eq. (35) can be written as

(38) $$\alpha c_{\mathrm{ss}}(1+{\left(\kappa_{m}(1+{\left(\frac{c_{\mathrm{ss}}}{\kappa_{c}}\right)}^{y})\right)}^{x})-\mu_{m}=0,$$

where

(39) $$\alpha =\frac{\delta_{m}\delta_{h}\delta_{c}}{\mu_{c}}.$$

Expanding, we find that the steady states are given by the solution of the polynomial equation of degree (xy+1),

(40) $$\frac{\beta }{\gamma^{x}}c_{\mathrm{ss}}^{\mathrm{yx}+1}+\frac{x\beta }{\gamma^{\left(x-1\right)}}c_{\mathrm{ss}}^{y(x-1)+1}\;+\cdots +\frac{x\beta }{\gamma }c_{\mathrm{ss}}^{y+1}+(\alpha +\beta )c_{\mathrm{ss}}-\mu_{m}=0,$$

where $\beta =\alpha \kappa_{m}^{x}$ and $\gamma =\kappa_{c}^{y}$. As all parameters are positive, we may apply the results of Descartes' Rule of Signs which states that the number of positive real roots of the system is either equal to the number of variations of signs in the coefficients of Eq. (40) or less than this by an even integer (Murray, 2002). As there is only one sign change in the sequence of coefficients Eq. (40), the system has only one positive real root, and therefore only one physiologically valid fixed point.

### 5.2 Fixed point stability

We now consider the stability of this fixed point by investigation of the eigenvalues of the linearised Jacobian matrix **J** of the system equations (30)–(32). The Jacobian is given by

(41) $$J=[\begin{array}{ccc} f_{m} & f_{h} & f_{c}\\ g_{m} & g_{h} & g_{c}\\ j_{m} & j_{h} & j_{c} \end{array}]=[\begin{array}{ccc} -\delta_{m} & 0 & -\psi \\ 1 & -\delta_{h} & 0\\ 0 & \mu_{c} & -\delta_{c} \end{array}],$$

with

(42) $$\psi =\frac{\mathrm{xy}\mu_{m}\kappa_{m}^{x}c_{\mathrm{ss}}^{y-1}{\left(1+{\left(\frac{c_{\mathrm{ss}}}{\kappa_{c}}\right)}^{y}\right)}^{x-1}}{\kappa_{c}^{y}{\left(1+\kappa_{m}^{x}{\left(1+{\left(\frac{c_{\mathrm{ss}}}{\kappa_{c}}\right)}^{y}\right)}^{x}\right)}^{2}}.$$

We note that ψ≥0 as all parameter values are positive and that $c_{\mathrm{ss}}\geq 0$ for physiologically valid parameter ranges.

Calculation of the eigenvalues of **J** requires the solution of the equation

(43) det(J−λI)=0,

where *λ* are the eigenvalues to be found and **I** is the identity matrix. Evaluation of Eq. (43) leads to the characteristic equation,

(44) $$\lambda^{3}+(\delta_{m}+\delta_{h}+\delta_{c})\lambda^{2}\;+(\delta_{m}\delta_{h}+\delta_{m}\delta_{c}+\delta_{h}\delta_{c})\lambda \;+(\delta_{m}\delta_{h}\delta_{c}+\mu_{c}\psi )=0,$$

which has three roots, the eigenvalues *λ*_1_, *λ*_2_ and *λ*_3_. Firstly we note that ψ≥0 ensures all coefficients of Eq. (44) are positive and thus by Descartes’ Rule of Signs there can be no purely positive real eigenvalues. There are then two cases for the roots of (44), either three negative real eigenvalues or one negative real eigenvalue and a pair of complex conjugate eigenvalues.

The fixed point is stable if and only if the real parts of *λ*_1_, *λ*_2_ and *λ*_3_ are negative. To determine for which conditions this occurs, we apply the Routh–Hurwitz Stability criteria to Eq. (44) (Murray, 2002). Routh–Hurwitz's criteria applied to a cubic equation of the form

$$\lambda^{3}+a_{2}\lambda^{2}+a_{1}\lambda +a_{0}=0$$

are satisfied if and only if $a_{0}>0$, $a_{1}>0$, $a_{2}>0$ and $a_{1}a_{2}-a_{0}>0$. That is, the necessary and sufficient condition for the roots of Eq. (44) to have negative real part requires

(45) $$\delta_{m}+\delta_{h}+\delta_{c}>0,$$

(46) $$\delta_{m}\delta_{h}+\delta_{m}\delta_{c}+\delta_{h}\delta_{c}>0,$$

(47) $$\delta_{m}\delta_{h}\delta_{c}+\mu_{c}\psi >0,$$

(48) $$(\delta_{m}+\delta_{h}+\delta_{c})(\delta_{m}\delta_{h}+\delta_{m}\delta_{c}+\delta_{h}\delta_{c})\;-(\delta_{m}\delta_{h}\delta_{c}+\mu_{c}\psi )=\rho (\delta_{m},\delta_{h},\delta_{c})>0.$$

Since all parameters are positive and real, conditions (45)–(47) hold. Thus the stability of the roots is dependent on condition (48). The possible dynamic behaviour of the system can be summarised as follows.

*Case* I: $\rho (\delta_{m},\delta_{h},\delta_{c})>0$. Here all real parts of all eigenvalues are negative. In this case the steady state is stable. This stable steady state may arise in one of two ways: (i) *Case* Ia: where all eigenvalues are real and negative. This will result in a stable node, where the concentrations of mRNA, protein and cholesterol will tend monotonically to a steady state; and (ii) *Case* Ib: where one eigenvalue is real and negative and two eigenvalues are complex conjugates with negative real part. In this case the fixed point is a stable spiral and the concentrations of mRNA, protein and cholesterol will demonstrate oscillatory convergence to a steady state.

*Case* II: $\rho (\delta_{m},\delta_{h},\delta_{c})=0$. By substituting this value of $\left(\delta_{m}\delta_{h}\delta_{c}+\mu_{c}\psi \right)$ into Eq. (44), we now have the characteristic equation given by

$$\lambda^{3}+\gamma_{1}\lambda^{2}+\gamma_{2}\lambda +\gamma_{1}\gamma_{2}=0,\;(\lambda +\gamma_{1})(\lambda^{2}+\gamma_{2})=0,$$

where we have $\gamma_{2}=(\delta_{m}\delta_{h}+\delta_{m}\delta_{c}+\delta_{h}\delta_{c})$ and $\gamma_{1}=(\delta_{m}+\delta_{h}+\delta_{c})$. Therefore the characteristic equation has two conjugate roots $\lambda_{1,2}$ on the imaginary axis and one negative real eigenvalue *λ*_3_ given by

(49) $$\lambda_{1,2}=\pm i\sqrt{\left(\delta_{m}\delta_{h}+\delta_{m}\delta_{c}+\delta_{h}\delta_{c}\right)},$$

(50) $$\lambda_{3}=-(\delta_{m}+\delta_{h}+\delta_{c})$$

one negative real eigenvalue and two pure imaginary eigenvalues. The existence of two conjugate eigenvalues on the imaginary axis means that the stability of the equilibrium cannot be directly determined; this case is discussed in detail in Section 6.1.

*Case* III: $\rho (\delta_{m},\delta_{h},\delta_{c})<0$. Here one eigenvalue is real and positive and two eigenvalues are complex conjugates with positive real part. In this case the steady state is unstable, implying that end product concentration would grow unboundedly. This case is biologically infeasible and hence we ignore it for the remainder of this paper.

## 6 Fixed point stability – variation of *μ*_*c*_

The eigenvalues of Eq. (44) can move between each case under the variation of system parameters. As an example we consider the effect of varying *μ*_*c*_ on the system dynamics. This parameter may be varied so that a pair of complex conjugate eigenvalues can either move into the negative real half plane (a stable focus equilibrium) or into the positive real half plane (an unstable focus equilibrium). The point where the eigenvalues cross the imaginary axis (Case II) occurs at a critical value of *μ*_*c*_ denoted by $\mu_{c}^{⁎}$. At this point a unique, closed periodic orbit may bifurcate locally from the equilibrium as it changes stability. The isolated, closed trajectory is known as a limit cycle and causes oscillatory behaviour. This phenomenon is called a Hopf bifurcation (Guckenheimer and Holmes, 1983) and its existence dictates that the concentrations of *m*, *h* and *c* will oscillate.

### 6.1 Hopf bifurcation existence

According to the Hopf bifurcation theorem (Guckenheimer and Holmes, 1983), a bifurcation occurs for a critical value $\mu_{c}=\mu_{c}^{⁎}$, for which the following two conditions are fulfilled, at the equilibrium point $\left(m_{\mathrm{ss}},h_{\mathrm{ss}},c_{\mathrm{ss}}\right)$:

1. The matrix **J** has two complex eigenvalues
  $$\lambda_{2,3}=\theta (\mu_{c})\pm i\omega (\mu_{c}),$$
  in some neighbourhood of $\mu_{c}^{⁎}$ and for $\mu_{c}=\mu_{c}^{⁎}$ these eigenvalues are purely imaginary, that is,
  $$\theta (\mu_{c}^{⁎})=0\;\mathrm{and}\;\omega (\mu_{c}^{⁎})\neq 0.$$
  This non-hyperbolicity condition is a necessary condition for the Hopf bifurcation.
2. The relation described by
  $${\frac{d\theta (\mu_{c})}{d\mu_{c}}|}_{\mu_{c}=\mu_{c}^{⁎}}\neq 0,$$
  holds in some neighbourhood of $\mu_{c}^{⁎}$. This is a sufficient condition for the Hopf bifurcation and is also known as the transversality or Hopf crossing condition. It ensures that the eigenvalues cross the imaginary axis with non-zero speed and thus ensures that the crossing of the complex conjugate pair at the imaginary axis is not tangent to the imaginary axis. If this is not the case we may observe, for example, the occurrence in which the eigenvalues move up to the imaginary axis and then reverse direction without crossing.

We notice that the first condition has already been shown to hold at the critical value $\mu_{c}^{⁎}$, given by the solution of

$$\mu_{c}^{⁎}=\frac{\left((\delta_{m}+\delta_{h}+\delta_{c})(\delta_{m}\delta_{h}+\delta_{m}\delta_{c}+\delta_{h}\delta_{c})-\delta_{m}\delta_{h}\delta_{c}\right)}{\psi },$$

where *ψ* is given by Eq. (42), together with the equation determining the equilibrium value of *c*_*ss*_ for $\mu_{c}^{⁎}$:

$$\frac{{\left(c_{\mathrm{ss}}\right)}^{\mathrm{yx}+1}}{{\left(\kappa_{c}^{y}\right)}^{x}}+x\frac{{\left(c_{\mathrm{ss}}\right)}^{y(x-1)+1}}{{\left(\kappa_{c}^{y}\right)}^{x-1}}+\cdots +x\frac{{\left(c_{\mathrm{ss}}\right)}^{y+1}}{\left(\kappa_{c}^{y}\right)}\;+(\frac{1}{\kappa_{m}^{x}}+1)c_{\mathrm{ss}}-(\frac{\mu_{m}}{\kappa_{m}^{x}\delta_{m}\delta_{h}\delta_{c}})\mu_{c}^{⁎}=0.$$

From the results of Case II we know that at this value of $\mu_{c}^{⁎}$ the characteristic polynomial Eq. (44) has two purely imaginary roots $\pm i\omega (\mu_{c}^{⁎})$, given in Eqs. (49) and (50), where

(51) $$\omega (\mu_{c}^{⁎})=\sqrt{\left(\delta_{m}\delta_{h}+\delta_{m}\delta_{c}+\delta_{h}\delta_{c}\right)}\neq 0.$$

To show that the second condition holds we use the Implicit Function Theorem. For each $\mu_{c}\in R$ and the corresponding system, Eqs. (30)–(32), define

$$k(\mu_{c},\lambda )=p(\lambda ),$$

as a function of two variables *μ*_*c*_ and *λ*, where p(λ) is the characteristic polynomial of the system equations (30)–(32) defined by Eq. (44).

Let the complex eigenvalues $\lambda (\mu_{c})=\theta (\mu_{c})\pm i\omega (\mu_{c})$ be the roots of the characteristic polynomial. Hence, for these eigenvalues we have

(52) $$k(\mu_{c},\lambda (\mu_{c}))=0,$$

where this represents an implicit function of two variables *μ*_*c*_ and *λ*. The Implicit Function Theorem tells us that we may define *μ*_*c*_ as a function of *λ* near the point $\left(\mu_{c}^{⁎},\lambda (\mu_{c}^{⁎})\right)$, and the derivative of this function is given by

(53) $${\frac{d\lambda }{d\mu_{c}}(\mu_{c}^{⁎})|}_{\mu_{c}=\mu_{c}^{⁎}}=-{\left(\left(\frac{\partial k}{\partial \mu_{c}}\right)/\left(\frac{\partial k}{\partial \lambda }\right))\right|}_{\mu_{c}=\mu_{c}^{⁎}},$$

providing

$$\frac{\partial k}{\partial \lambda }\neq 0.$$

We begin by computing the derivative of the function $k(\mu_{c},\lambda (\mu_{c}))$ with respect to *λ*, and evaluating this at the critical point $\mu_{c}^{⁎}$. Thus we have,

$${\frac{\partial k}{\partial \lambda }(\mu_{c},\lambda )|}_{\left(\mu_{c},\lambda )=(\mu_{c}^{⁎},\pm i\omega (\mu_{c}^{⁎})\right)},\;=3\lambda^{2}+2(\delta_{m}+\delta_{h}+\delta_{c})\lambda \;{+(\delta_{m}\delta_{h}+\delta_{m}\delta_{c}+\delta_{h}\delta_{c})|}_{(\mu_{c},\lambda )=(\mu_{c}^{⁎},\pm i\omega (\mu_{c}^{⁎})),}\;=3{\left(\pm i\omega (\mu_{c}^{⁎})\right)}^{2}+2(\delta_{m}+\delta_{h}+\delta_{c})(\pm i\omega (\mu_{c}^{⁎}))\;+(\delta_{m}\delta_{h}+\delta_{m}\delta_{c}+\delta_{h}\delta_{c}).$$

Simplifying in conjunction with the fact that $\omega^{2}(\mu_{c}^{⁎})=(\delta_{m}\delta_{h}+\delta_{m}\delta_{c}+\delta_{h}\delta_{c})$ from Eq. (51), we obtain

(54) $$\frac{\partial k}{\partial \lambda }=-2\omega^{2}(\mu_{c}^{⁎})\pm 2i(\delta_{m}+\delta_{h}+\delta_{c})\omega (\mu_{c}^{⁎})\neq 0.$$

Furthermore, from the characteristic polynomial Eq. (44), we have

(55) $${\frac{\partial k}{\partial \mu_{c}}(\mu_{c},\lambda )|}_{\left(\mu_{c},\lambda )=(\mu_{c}^{⁎},\pm i\omega (\mu_{c}^{⁎})\right)}=\psi ,$$

where, we have previously noted that ψ≥0. However, in the case *ψ*=0, the Jacobian matrix becomes

$$J=[\begin{array}{ccc} -\delta_{m} & 0 & 0\\ 1 & -\delta_{h} & 0\\ 0 & \mu_{c} & -\delta_{c} \end{array}],$$

which is lower triangular and hence has three negative real eigenvalues given by the entries of the leading diagonal, specifically $-\delta_{m},-\delta_{h}$ and $-\delta_{c}$. This violates the requirement of condition 1 that the matrix **J** has two complex eigenvalues. Therefore we can conclude that in the case of a Hopf bifurcation, ψ≠0 and we need only be concerned with the strict inequality ψ>0.

Eqs. (54) and (55) together with Eq. (53) yield

$$\frac{d\lambda }{d\mu }(\mu_{c}^{⁎})=\frac{1}{2\omega (\mu_{c}^{⁎})}(\frac{\psi }{-\omega (\mu_{c}^{⁎})\pm i(1+\delta_{h}+\delta_{c})}).$$

Upon the rationalisation of the denominator of this complex fraction we obtain

$$\frac{d\lambda }{d\mu_{c}}(\mu_{c}^{⁎})=\frac{1}{2\omega (\mu_{c}^{⁎})}(\frac{-\omega (\mu_{c}^{⁎})\psi }{\omega^{2}(\mu_{c}^{⁎})+{\left(\delta_{m}+\delta_{h}+\delta_{c}\right)}^{2}})+\frac{i}{2\omega (\mu_{c}^{⁎})}(\frac{\mp \psi (\delta_{m}+\delta_{h}+\delta_{c})}{\omega^{2}(\mu_{c}^{⁎})+{\left(\delta_{m}+\delta_{h}+\delta_{c}\right)}^{2}}),$$

and since ψ>0,

$${\frac{d\theta (\mu_{c})}{d\mu_{c}}|}_{\mu_{c}=\mu_{c}^{⁎}}=Re(\frac{d\lambda }{d\mu_{c}}(\mu_{c}^{⁎}))\;=\frac{1}{2}(\frac{-\psi }{\omega^{2}(\mu_{c}^{⁎})+{\left(\delta_{m}+\delta_{h}+\delta_{c}\right)}^{2}})<0,$$

and the second condition of the Hopf theorem is fulfilled. Thus we have proved the existence of a Hopf bifurcation at the critical value $\mu_{c}=\mu_{c}^{⁎}$.

### 6.2 Hopf bifurcation stability

Just as the steady states of a system may be stable or unstable, the limit cycle which branches from the fixed point in a Hopf bifurcation may also be stable or unstable. A stable limit cycle occurs if the Hopf bifurcation is supercritical whereas an unstable limit cycle is the product of a subcritical bifurcation.

At a subcritical bifurcation a unique and unstable limit cycle, which exists for $\mu_{c}<\mu_{c}^{\times }$, is absorbed by a stable spiral equilibrium. The equilibrium becomes unstable for $\mu_{c}>\mu_{c}^{\times }$; in this case diverging oscillations and therefore unbounded growth in the evolution of variables is seen. In a supercritical bifurcation the equilibrium point prior to the Hopf bifurcation is a stable spiral, and concentrations of mRNA, protein and cholesterol display oscillatory decay before reaching a steady state value. At the bifurcation point $\mu_{c}=\mu_{c}^{\times }$, a limit cycle is born. At this point the equilibrium changes stability and becomes unstable. For $\mu_{c}>\mu_{c}^{\times }$, this becomes a unique and stable small amplitude limit cycle; here the concentrations of mRNA, protein and cholesterol exhibit stable oscillations.

As the limit cycle is stable, any small perturbation from the closed trajectory causes the system to return to the limit cycle resulting in self sustained oscillations in concentrations of mRNA, protein and cholesterol within the region of some equilibrium value. Thus, as the occurrence of a supercritical Hopf bifurcation will result in behaviour which is analogous to the physiological process of homeostasis, it is necessary to determine the stability of the Hopf bifurcation. This analysis was undertaken as follows.

Numerical solutions to Eqs. (30)–(32) were obtained using MATLAB (MATLAB, 8.0.0.783, The MathWorks Inc., Natick, MA, 2012) and the characteristics of system bifurcations and limit cycles were explored using the MATLAB numerical continuation toolbox Matcont (Dhooge et al., 2003). The basic principle of this toolbox is to consider a system of ODEs

(56) $$\frac{dx}{\mathrm{dt}}=f(x,\mu )\;x\in R^{n},\;\mu \in R^{1},$$

with an equilibrium point at $\left(x_{0},\mu_{0}\right)$. The principle of numerical continuation requires finding a solution curve *σ* of $f(x_{0},\mu_{0})=0$ with $\sigma (0)=(x_{0},\mu_{0})$ which describes how the equilibrium point varies. The curve *σ* is traced by means of a predictor-corrector algorithm and bifurcations along *σ* are detected using a suitable test function which changes sign at the bifurcation point.

Once the Hopf bifurcation has been detected, Matcont calculates the stability of the subsequent limit cycle by calculating the first Lyapunov coefficient or Lyapunov characteristic exponent, $l_{1}(0)$, of the dynamical system near the bifurcation point. This coefficient describes the average rate at which neighbouring trajectories in the phase space converge or diverge. Specifically,

• $l_{1}(0)<0$ implies that the system is attracted to a stable periodic orbit and
• $l_{1}(0)>0$ implies that the system is attracted to an unstable periodic orbit.

In the case of Eqs. (30)–(32) with *μ*_*c*_ as the bifurcation parameter, we find that a Hopf bifurcation is predicted to occur at the point ($\mu_{c}^{\times }$, *c*^⁎^)=(1.809, 0.011), whose values are the solution of the simultaneous equations (40) and (48). This bifurcation has a negative first Lyapunov coefficient which indicates that a stable limit cycle is produced and the bifurcation is supercritical.

The results of the Hopf bifurication existence and stability analysis of the governing system of Eqs. (30)–(32) can now be discussed in the context of cellular cholesterol homeostasis. Homeostasis is the tendency of a system to regulate its internal environment by maintaining a stable condition. All homeostatic mechanisms use feedback inhibition to facilitate a constant level. Essentially this involves controlling the concentration of a particular variable within a narrow range in the region of an optimal value. If this concentration alters, the feedback inhibition pathway automatically initiates a corrective mechanism which reverses this change and brings it back towards an equilibrium. In a system controlled by feedback inhibition, the equilibrium is never perfectly maintained, but constantly oscillates about an optimal level. Thus the existence of the Hopf bifurcation and the consequent appearance of small amplitude oscillations in the concentrations of mRNA, protein and cholesterol, are significant in its similarity to the behaviour of a homeostatic system.

### 6.3 Illustration of system behaviour

In this section we present numerical solutions to Eqs. (30)–(32) using the MATLAB stiff differential equation solver ODE15s (MATLAB, 8.0.0.783, The MathWorks Inc., Natick, MA, 2012) for various values of *μ*_*c*_ to illustrate the system behaviour elucidated in the previous sections. All remaining parameters were held constant as detailed in Table 1. The parameter *μ*_c_ was varied between 1.53×10^−2^ s^−1^ and 6.46×10^−2^ s^−1^ (physiologically valid limits) to demonstrate the variation of system behaviour through Cases I to II.

**Fig. 3:**
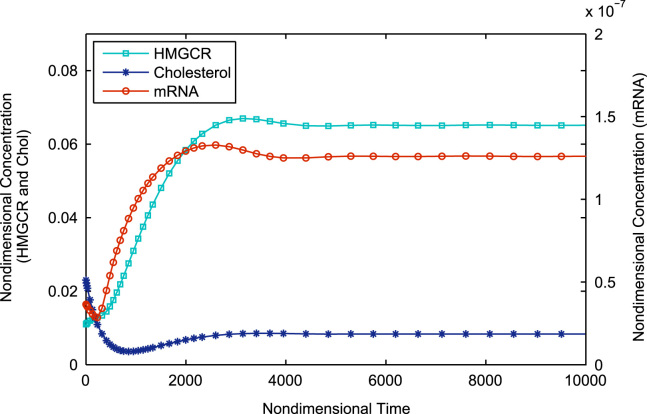
Stable node equilibrium (corresponding to Case Ia) where there are three negative real eigenvalues; variable concentrations exhibit monotonic convergence towards a steady state value. All parameters are as in Table 2 except nondimensional *μ*_*c*_=0.462. Nondimensional initial conditions: *m*(0)=3.65×10^−8^, *h*(0)=1.10×10^−5^ and *c*(0)=2.30×10^−2^. Note that the evolution of HMGCR has been rescaled to allow for easier comparison.

**Fig. 4:**
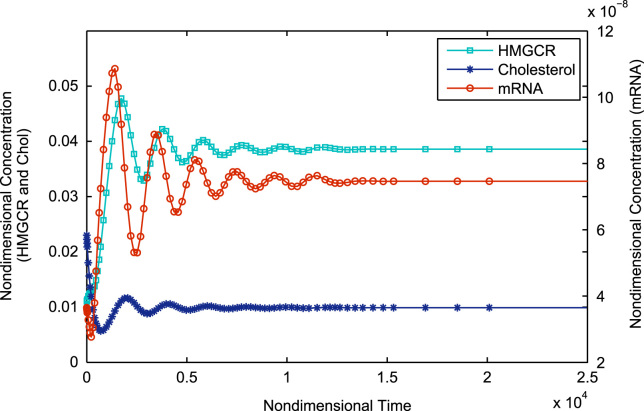
Stable spiral equilibrium (corresponding to Case Ib) where there is one negative real eigenvalue and a pair of complex conjugate eigenvalues with negative real part; variable concentrations exhibit oscillatory convergence towards a steady state value. Initial conditions are as in Fig. 3. All parameters are as in Table 2 except for *μ*_*c*_ which has been increased 2 fold to *μ*_*c*_=0.923.

**Fig. 5:**
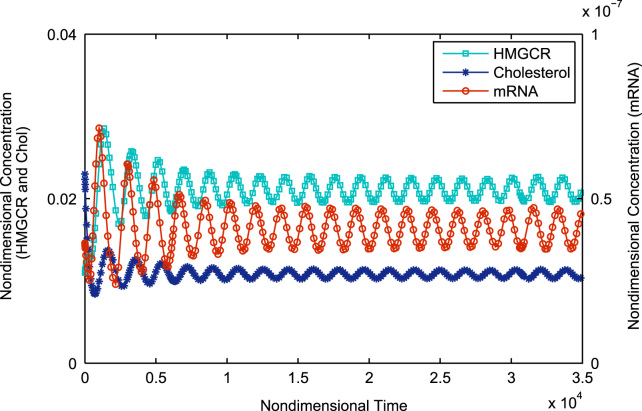
Following the occurrence of a Hopf bifurcation the (now unstable) equilibrium is attracted to a stable limit cycle. Variable concentrations exhibit purely oscillatory behaviour; the oscillations are stable. Initial conditions are as in Fig. 3. All parameters are as in Table 2 except for *μ*_*c*_ which has been increased approximately 4 fold to *μ*_*c*_=1.946.

Simulation of Eqs. (30)–(32) starting with the initial value of 1.53×10^−2^ s^−1^ shows monotonic non-oscillatory convergence to a steady state, equivalent to Case Ia as illustrated in Fig. 3. Continually increasing *μ*_*c*_ shows the system shifting to Case Ib. Thus we see oscillatory convergence to a steady-state as shown in Fig. 4. Still further increases in *μ*_*c*_ see the system reaching Case II, where we have pure oscillations in mRNA, HMGCR and cholesterol; this is illustrated in Fig. 5. The Hopf bifurcation occurs at the transition from Case Ib to Case II.

Following the bifurcation, the evolution of the concentrations of mRNA, HMGCR and cholesterol are purely periodic, with small amplitude stable oscillations. The period of the oscillations in Fig. 5 is approximately 16.9 h; further numerical investigations have revealed that on manipulation of system parameters, the oscillatory period can vary between approximately 12 and 24 h.

We also find that after *μ*_*c*_ has passed through its critical Hopf bifurcation value, no further changes in dynamical behaviour occur. That is, once the system becomes oscillatory, it remains in this manner for all $\mu_{c}>\mu_{c}^{\times }$.

## 7 Remaining parameter analysis and system behaviour

Further numerical investigation of the governing system of equations has shown that each of the system parameters, if varied whilst all other parameters are kept constant, are capable of inducing a Hopf bifurcation. In the case of synthesis rates, *μ*_*m*_ and *μ*_*c*_, only one Hopf bifurcation occurs and is supercritical. Any oscillatory behaviour the system demonstrates is always stable and these oscillations persist for any parameter value greater than its critical bifurcation value.

We note, however, that if either the degradation rates, $\left(\delta_{m},\delta_{h},\delta_{c}\right)$, or binding affinities $\left(\kappa_{m},\kappa_{c}\right)$, are taken to be bifurcation parameters, qualitatively different behaviour from the case discussed above is seen. Calculation of the critical values for these parameters indicates that there are two physiologically valid points where a Hopf bifurcation may occur.

Examining the case of *δ*_*c*_ we see that the critical value $\delta_{c}^{⁎}$ for which a Hopf bifurcation may occur is given by the solution of the equation

(57) $$(\delta_{m}+\delta_{h}){\left(\delta_{c}^{⁎}\right)}^{2}+{\left(\delta_{m}+\delta_{h}\right)}^{2}\delta_{c}^{⁎}\;+(\delta_{m}^{2}\delta_{h}+\delta_{m}\delta_{h}^{2}-\mu_{c}\psi )=0,$$

which is quadratic in $\delta_{c}^{⁎}$ and hence, for the case of two positive real roots, gives rise to the possibility that there are two Hopf bifurcation points associated with this parameter. This result in turn affects the steady-states of *c*_*ss*_ which are determined from

$$\delta_{c}^{⁎}\frac{{\left(c_{\mathrm{ss}}\right)}^{\mathrm{yx}+1}}{{\left(\kappa_{c}^{y}\right)}^{x}}+x\delta_{c}^{⁎}\frac{{\left(c_{\mathrm{ss}}\right)}^{y(x-1)+1}}{{\left(\kappa_{c}^{y}\right)}^{x-1}}+\cdots +x\delta_{c}^{⁎}\frac{{\left(c_{\mathrm{ss}}\right)}^{y+1}}{\left(\kappa_{c}^{y}\right)}\;+(\frac{\delta_{c}^{⁎}}{\kappa_{m}^{x}}+\delta_{c}^{⁎})c_{\mathrm{ss}}-(\frac{\mu_{m}\mu_{c}}{\kappa_{m}^{x}\delta_{m}\delta_{h}})\mu_{c}^{⁎}=0.$$

The eigenvalues at this critical point are given by

(58) $$\lambda_{1,2}=\pm i\sqrt{\left(\delta_{h}+\delta_{c}+\delta_{h}\delta_{c}^{⁎}\right)},$$

(59) $$\lambda_{3}=-(1+\delta_{h}+\delta_{c}^{⁎}),$$

and so the first Hopf bifurcation condition holds. Proceeding in the manner of the calculation for $\mu_{c}^{⁎}$, we find

(60) $${\frac{d\theta (\delta_{c})}{d\delta_{c}}|}_{\delta_{c}=\delta_{c}^{⁎}}=Re\frac{d\lambda }{d\delta_{c}}(\delta_{c}^{⁎})=\frac{\omega^{2}(\delta_{c}^{⁎})+(1+\delta_{h})(1+\delta_{h}+\delta_{c}^{⁎})-\delta_{h}}{2(\omega^{2}(\delta_{c}^{⁎})+{\left(1+\delta_{h}+\delta_{c}^{⁎}\right)}^{2})}>0,$$

and therefore the second condition of the Hopf theorem also holds.

**Fig. 6:**
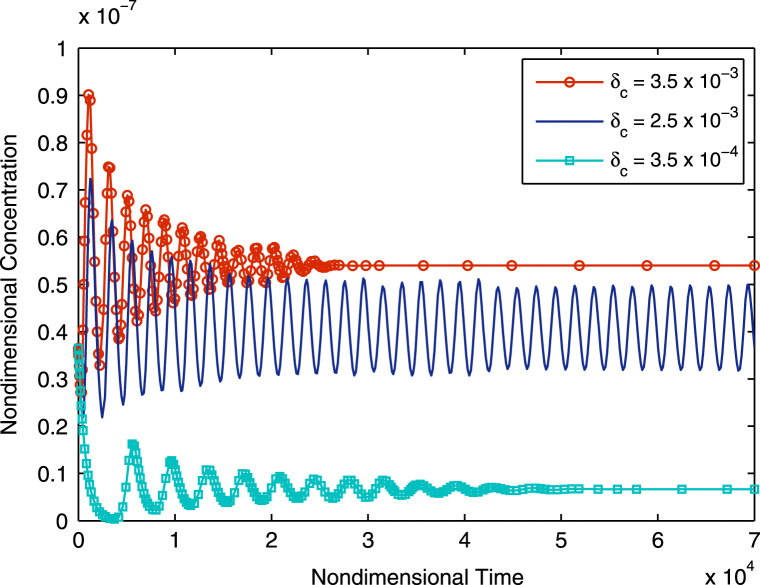
The response of mRNA concentration to variation of *δ*_*c*_. A clearly demarcated region of purely stable oscillatory behaviour is visible between stable steady state solutions. All parameters are as in Table 2 except for *δ*_*c*_ which is successively varied with nondimensional values as indicated. Initial conditions are as in Fig. 3.

Here, the unique equilibrium value undergoes two Hopf bifurcation points. Before the first of these points, the equilibrium is a stable spiral. At the first bifurcation point a supercritical Hopf bifurcation leads to the appearance of a stable periodic orbit (as the eigenvalues of the system cross the imaginary axis from left to right). The amplitude of this limit cycle increases initially as *δ*_*c*_ increases whilst later decreasing until the second bifurcation point where the limit cycle disappears (as the eigenvalues of the system cross the imaginary axis from right to left) and the equilibrium point becomes stable again. Numerical analysis demonstrates a negative first Lyapunov coefficient for Hopf bifurcations confirming their supercriticality. For any value of *δ*_*c*_ falling between the two bifurcation values purely oscillatory behaviour is generated, whilst outside this region only stable non-oscillatory solutions exist as illustrated in Fig. 6.

## 8 Discussion

We have formulated and solved a deterministic ODE model of cholesterol biosynthetic regulation by SREBP-2 in a hepatocyte. The model predicts the existence of oscillatory behaviour within the system which we believe is important in understanding cholesterol homeostasis. In the HMGCR system, such a mechanism means that perturbations may be made to certain system variables without losing the required concentration within which cholesterol is allowed to vary to guard against cytotoxicity. Other advantages to this dynamic mechanism include limiting the time during which cholesterol concentration is necessarily elevated within the cell in response to increased demand. Furthermore, controlling cellular cholesterol levels in this manner may incur less demand on cellular energy supplies than sustained elevation. Dynamic oscillatory steady-state behaviour allows the system to vary between upper and lower bounds consistent with an oscillatory homeostatic mechanism (Ghosh and Chance, 1964; Waxman et al., 1966).

Following the work of Goodwin (1965) and Griffith (1968) negative feedback regulation of mRNA levels, which are modulated by end product concentration, are often modelled using a Hill type function such that the

$$\frac{\mathrm{dm}}{\mathrm{dt}}=\frac{\mu }{K^{n}+b^{n}}-\alpha m,$$

where m=m(t) is the mRNA concentration, b=b(t) is the concentration of the end product, *μ* is the rate of mRNA transcription, *K* is the Hill constant, *n* is the Hill coefficient and *α* is the rate of mRNA degradation. Goodwin (1965) and Griffith (1968) showed that such a system will exhibit oscillations should n≥8. Values of *n* greater than approximately 4 are, however, deemed biologically implausible. In comparison, our mathematical model formulation has explicitly accounted for the interaction between not only the transcriber (in this case SREBP-2), but the negative regulation of the transcriber by the end product (cholesterol). The form of Eq. (24) accurately accounts for these interactions and allows biologically realistic values for them to be included in the model formulation whilst accurately accounting for the system dynamics.

Whilst our mathematical model has been formulated in the context of cholesterol biosynthesis this specific process of transcription factor regulated gene expression could be applicable to other pathways regulated by SREBP-2, in addition to the modulation of other lipid regulating proteins by the SREBP-1a and SREBP-1c isoforms. Further experimental research is necessary to evaluate these mathematical results and to clarify the system behaviour. However, this model and its analysis may serve as a basis for the investigation of transcription factor mediated gene expression dynamics, and furthermore constitute an important component of the synthetic engineering of biological circuits (Zhang and Jiang, 2010).

## Figures and Tables

**Table 1:** Summary of model parameter values. Details of parameter derivations are given in Appendix A.

Parameter	Description	Dimensional value
${\overset{¯}{\mu }}_{m}$	HMGCR transcription rate	5.17×10^5^ molecules ml^−1^ s^−1^
${\overset{¯}{\mu }}_{h}$	HMGCR translation rate	3.33×10^−2^ s^−1^
${\overset{¯}{\mu }}_{c}$	Cholesterol synthesis rate	4.33×10^−2^ s^−1^
${\overset{¯}{\delta }}_{m}$	HMGCR mRNA degradation rate	4.48×10^−5^ s^−1^
${\overset{¯}{\delta }}_{h}$	HMGCR degradation rate	6.42×10^−5^ s^−1^
${\overset{¯}{\delta }}_{c}$	Cholesterol utilisation rate	1.20×10^−4^ s^−1^
${\overset{¯}{\kappa }}_{m}$	SREBP-2-HMGCR gene dissociation constant	$O(10^{13})$ molecules ml^−1^
${\overset{¯}{\kappa }}_{c}$	SREBP-2-Cholesterol dissociation constant	$O(10^{14})$ molecules ml^−1^
*x*	Molecules of SREBP-2 binding to gene	3
*y*	Molecules of cholesterol binding to SREBP-2	4

**Table 2:** Non-dimensional parameter values.

Parameter	Description	Nondimensional value
*μ*_*c*_	Cholesterol synthesis rate	1.30
*μ*_*m*_	HMGCR transcription rate	1.90×10^−10^
*δ*_*m*_	HMGCR mRNA degradation rate	1.35×10^−3^
*δ*_*h*_	HMGCR degradation rate	1.93×10^−3^
*δ*_*c*_	Cholesterol utilisation rate	3.60×10^−3^
*κ*_*m*_	SREBP-2-HMGCR gene dissociation constant	1.00×10^−4^
*κ*_*c*_	SREBP-2-cholesterol dissociation constant	1.00×10^−3^

## Appendix A Parameter derivation and estimation

### A.1 Determination of synthesis parameters

Information on the rates of transcription and translation are rarely quantified in terms of mass per unit time, instead these rates are often measured relative to a control process. Therefore in order to estimate practical values for these parameters, we consider the relevant biological mechanisms.

#### A.1.1 Rate of HMGCR mRNA transcription – ${\overset{¯}{\mu }}_{m}$

In order to produce an estimate for the transcription rate the assumption that any time delays involved in the initiation of transcription and promoter clearance are negligible, is made. It is also assumed that no abortive transcripts are produced. Liver cells are somatic and therefore the majority are diploid, meaning they contain two chromosomes and thus normal amounts of DNA. Ignoring the existence of both tetraploidy and double nuclei that can be present within some liver cells, we assume all liver cells to be diploid.

We estimate the rate of transcription in terms of nucleotides per unit time in a typical mammalian gene. It is possible for a cell to transcribe 14,000 base pairs in 20 min giving a rate of 12 bases per second (Darzacq et al., 2007). This value is used to provide a rough estimate of the rate of transcription, equivalent to the number of mRNA molecules produced per cell per unit time.

The human HMG-CoA reductase gene is 24,826 bases long (Goldstein and Brown, 1984). To transcribe one molecule of primary HMG-CoA reductase mRNA, from one gene, assuming a rate of 12 bases per second, takes 2068.83 s. We then take into account that the post transcriptional processing steps of mRNA cleavage, 5' capping and polyadenylation are rate limiting. A delay of approximately 30 min has been added to account for this. Therefore an approximation of the time it takes to transcribe one molecule of primary HMG-CoA reductase mRNA from one gene is 3868.83 s. Per gene, this equates to

(A.1) $$2.59\times 10^{-4}\;\mathrm{molecules}\;\mathrm{HMGCR}\;\mathrm{mRNA}\;s^{-1}.$$

Therefore one gene can synthesise 2.59×10^−4^ molecules of HMG-CoA reductase mRNA per second. Taking diploidy into account there are 5.17×10^−4^ molecules HMG-CoA reductase mRNA being synthesised per cell per second.

Given an average cell volume of 1pl (1×10^−12^ l=1×10^−9^ ml), the required approximate rate of HMGCR transcription is given by

(A.2) $${\overset{¯}{\mu }}_{m}=\frac{5.17\times 10^{-4}\;{\text{moleculess}}^{-1}}{1\times 10^{-9}\;\mathrm{ml}}=5.17\times 10^{5}\;{\text{moleculesml}}^{-1}\;s^{-1}.$$

#### A.1.2 Rate of HMGCR protein translation – ${\overset{¯}{\mu }}_{h}$

To calculate an estimate of the rate of translation the following assumptions are made. Firstly, any effects caused by the transport of mRNA from the nucleus to its localisation in the cytoplasm are ignored. Also ignored are the effects of protein folding on transcriptional regulation as well as biochemical interactions amongst proteins. Finally any time delays in the elongation phase of protein synthesis are considered to be negligible.

The *in vivo* estimate from Slobin (1991) suggests that the translation rate for eukaryotic cells is 6 amino acids per second, where one amino acid is encoded by 3 nucleotides or bases. Many ribosomes can translate the same mRNA molecule simultaneously (Granner and Weil, 2006). Because of their large size, ribosomes cannot attach to mRNA any closer than 35 nucleotides apart. This detail allows estimation of the rate of transcription, equivalent to proteins synthesised per unit time from mRNA.

A human HMG-CoA reductase mRNA transcript contains 4475 bases (Goldstein and Brown, 1984). For one ribosome to transcribe one molecule of HMG-CoA reductase protein, from one HMG-CoA reductase mRNA, assuming a translation rate of 6 amino acids per second, where three bases code for one amino acid, takes

(A.3) $$\frac{4475\;\mathrm{amino}\;\mathrm{acids}}{18\;\mathrm{amino}\;\mathrm{acids/s}}=248.61\;s.$$

Taking into account that the rate limiting step in translation is the initiation of the process itself, a delay of approximately 60 min is added. This gives the approximation that each ribosome takes 3848.61 s to translate one molecule of HMG-CoA reductase from HMG-CoA reductase mRNA. Then per ribosome, this equates to

(A.4) $$\frac{1\;\mathrm{molecule}}{3848.61\;s}=2.598\times 10^{-4}\;{\text{moleculess}}^{-1}.$$

Thus, 2.598×10^−4^ molecules of HMG-CoA reductase protein may be synthesised per second per ribosome.

Given that the coding region of the HMG-CoA reductase mRNA is 4475 nucleotides long and a ribosome can attach every 35 nucleotides, 127.85 ribosomes can attach per mRNA molecule. Therefore, per HMG-CoA reductase mRNA molecule,

(A.5) $$2.598\times 10^{-4}\;{\text{moleculess}}^{-1}\;{\mathrm{ribosome}}^{-1}\;\times 127.85\;\mathrm{ribosomes}\;=3.32\times 10^{-2}\;{\text{moleculess}}^{-1},$$

that is, there are 3.32×10^−2^ molecules of HMG-CoA reductase protein being synthesised per second. We thus take

$${\overset{¯}{\mu }}_{h}=3.32\times 10^{-2}\;s^{-1}$$

as an approximation of the rate of HMGR translation.

#### A.1.3 HMGCR activity – ${\overset{¯}{\mu }}_{c}$

The value of 52 pmol mevalonate formed per minute per mg protein for HMGCR activity in human liver microsomes is taken directly from Tanaka et al. (1982). This must now be converted into a turnover number for HMGCR which is the maximum number of substrate molecules that an enzyme can convert to product per mole of catalytic site of the enzyme per unit time. The activity of an enzyme is the moles of substrate converted per unit time and is a measure of the quantity of active enzyme present. The specific activity is the activity of an enzyme per mg of total protein, i.e. a measure of enzyme efficiency.

HMG-CoA reductase is a tetrameric protein, composed of monomers arranged in two dimers. Each dimer has two active sites (Istvan et al., 2000) and has a molecular mass=99,906 Da. (The Dalton, (Da), (alternatively *atomic mass unit*) is a unit of mass used to express atomic and molecular masses. It is defined so that one mole of a substance with atomic or molecular mass 1 Da will have a mass of precisely 1 g.)

Therefore the full HMG-CoA reductase molecule is an enzyme containing 4 active sites with a molecular mass *M*_*r*_=2×99,906=199,812 Da, i.e. 1 mole HMG-CoA reductase has a mass of 199,812 g. The reaction catalysed by HMG-CoA reductase is given by

(A.6) $$\mathrm{HMG-CoA}+2\mathrm{NADPH}+2H^{+}\;⟶\mathrm{mevalonate}+2{\mathrm{NADP}}^{+}+\mathrm{CoASH},$$

which indicates a stoichiometry of one mole mevalonate being produced from one mole HMG-CoA. (Note all other substrates of this reaction are assumed to be present in excess).

Segel (1993) suggests that there are approximately 1000 different enzymes in a cell. Thus the moles of enzyme in 1 mg of protein are equivalent to

(A.7) $$(\frac{1\times 10^{-3}g}{199,812\;{g\;\mathrm{mol}}^{-1}}\times 1000)=5.00\times 10^{-12}\;\mathrm{mol},$$

and given 4 active sites per HMG-CoA reductase enzyme, there are 2.00×10^−11^ moles of enzyme active sites in 1 mg of protein. Given the specific activity of an enzyme, 52×10^−12^ mol min^−1^ mg^−1^, we have

(A.8) $$\frac{52\times 10^{-12}\;\mathrm{mol}\;\min^{-1}\;{\mathrm{mg}}^{-1}}{2.00\times 10^{-11}\;{\text{molmg}}^{-1}}=2.60\;\min^{-1}.$$

And so, an approximation to the turnover number for HMG-CoA reductase is given by

(A.9) $${\overset{¯}{\mu }}_{c}=4.33\times 10^{-2}\;s^{-1}.$$

### A.2 Determination of degradation rates – ${\overset{¯}{\delta }}_{m}$, ${\overset{¯}{\delta }}_{h}$ and ${\overset{¯}{\delta }}_{c}$

The parameters *δ*_*m*_ and *δ*_*h*_ determine the degradation rate of the HMGCR mRNA, and HMGCR enzyme molecule respectively. The calculation of these values is based on the half-lives, $t_{1/2}$, of the molecules as derived from an exponential decay model. For a decay constant *δ*, the rate of degradation of the variable is given by

(A.10) $$\delta =\frac{\ln\;2}{t_{1/2}}.$$

Data from Wilson and Deeley (1995) give a half-life of $t_{1/2}=4.3$ h for HMG-CoA reductase mRNA, measured in Hep G2 cells and so

(A.11) $${\overset{¯}{\delta }}_{m}=\frac{\ln\;2}{15,480\;s}=4.48\times 10^{-5}\;s^{-1}.$$

Data from Brown et al. (1974) give a half-life of $t_{1/2}=3$ h for the HMG-CoA reductase protein, measured in human fibroblast cells and so

(A.12) $${\overset{¯}{\delta }}_{h}=\frac{\ln\;2}{10,800\;s}=6.42\times 10^{-5}\;s^{-1}.$$

The parameter ${\overset{¯}{\delta }}_{c}$ determines the effective loss of cholesterol from the system as cholesterol does not degrade in a similar manner to the other species in the system. The value of this parameter may exhibit significant variation dependent on cellular conditions. For an initial value to be used in simulation, this parameter is assumed to be slightly faster than the degradation rates of protein and mRNA and ${\overset{¯}{\delta }}_{c}=1.20\times 10^{-4}\;s^{-1}$ is used.

### A.3 Determination of binding affinity parameters – ${\overset{¯}{\kappa }}_{m}$ and ${\overset{¯}{\kappa }}_{c}$

The parameter defining the binding reaction between SREBP and the HMGCR gene is *κ*_*m*_. As no specific experimental data is available for the binding affinity between SREBP and the SRE of the HMGCR gene, we use the results of Yang and Swartz (2011) who quantify the DNA binding affinity for other regulatory transcription factors. Their results provide an estimate of DNA transcription factor binding affinity in the region of 54.2 nmol; these estimates are in keeping with the hypothesis that the interaction between SREBPs and SREs is characterised by relatively high affinity and low saturation kinetics (Amemiya-Kudo et al., 2002). To convert these affinities into units of molecules per ml the following calculation is used.

A binding affinity of 54.2 nmol is equivalent to

(A.13) $$\frac{54.2\times 10^{-9}}{1000}\;\mathrm{moles/ml}\times N_{A}$$

(A.14) $$\;=3.26\times 10^{13}\;\mathrm{molecules/ml},$$

and so *κ*_*m*_ is taken to be $O(10^{13})$.

The parameter which describes the binding of cholesterol to SREBP is *κ*_*c*_. Experimental investigations with cholesterol on the SSD contained in SCAP have revealed difficulty in calculating kinetic constants for this interaction owing to the insolubility of both cholesterol and SCAP, and the necessity of performing kinetic experiments in micelles (Radhakrishnan et al., 2004). Data from Radhakrishnan et al. (2004) indicates that the binding reaction between cholesterol and SCAP is saturable and half-maximal binding occurs at approximately 100 nmol. This value is converted into units of molecules per ml using the following calculation.

100 nmol is equivalent to

(A.15) $$\frac{100\times 10^{-9}}{1000}\;\mathrm{moles/ml}\times N_{A}$$

(A.16) $$\;=6.02\times 10^{13}\;\mathrm{molecules/ml}.$$

and so *κ*_*c*_ is taken to be $O(10^{14})$.

### A.4 Binding coefficients – *x* and *y*

The work of Vallett et al. (1996) suggests that three binding locations on the HMGCR gene are available to SREBP and hence *x*=3. Experimental investigations on the SSD contained in SCAP have revealed its tetrameric nature (Radhakrishnan et al., 2004, 2008). That is, four molecules of cholesterol may bind to a SCAP–SREBP complex in order to promote inactivation and *y*=4.

### A.5 Concentration of total transcription factor – ${\overset{¯}{s}}_{0}$

In general, transcription factors have low gene expression, and are therefore present in relatively low concentrations within the cell (Sanguinetti et al., 2006). Since the SREBP signalling pathway has been simplified it is necessary to make some assumptions to obtain an estimate for this value. The majority of cellular cholesterol is located within the plasma membrane of the cell. Its concentration is governed by multiple regulatory proteins regulated in the ER which are themselves under the control of a small regulatory pool of ER cholesterol (Lange et al., 1999). The concentration of transcription factor, ${\overset{¯}{s}}_{0}$, is assumed to be of the order of this concentration, which allows small changes in the cellular cholesterol pool to be magnified in the transcription factor pathway. Lange et al. (1999) give an average of 0.45 nmol ER cholesterol per mg cell protein.

Avogadro's number $\left(N_{A}=6.022\times 10^{23}\right)$ and the assumption that one cell has approximately 300 pg of cell protein and a volume of 1 pl (1 mg of cell protein is thus equivalent to 3.3×10^−3^ ml) produces

(A.17) $${\overset{¯}{s}}_{0}=\frac{0.45\times 10^{-9}\;\mathrm{mol}\times 6.022\times 10^{23}\;{\text{moleculesmol}}^{-1}}{3.3\times 10^{-3}\;\mathrm{ml}},=8.21\times 10^{16}\;{\text{moleculesml}}^{-1}.$$
